# Supplementary material for: A high-resolution mRNA expression time course of embryonic development in zebrafish
Source: eLife. 2017 Nov 16;6:e30860. doi: 10.7554/eLife.30860 (PMC5690287; doi:10.7554/eLife.30860)
Supplement: Supplementary file 6. [file elife-30860-supp6.zip › biolayout-clusters-files/Cluster108-genes.html]

Cluster108


# Cluster108: Genes

| Ensembl ID | Gene Name | Chr | Start | End | Biotype |
| --- | --- | --- | --- | --- | --- |
| ENSDARG00000054533 | araf | 5 | 26254806 | 26269239 | protein\_coding |
| ENSDARG00000076895 | flrt3 | 13 | 33685826 | 33696534 | protein\_coding |
| ENSDARG00000006219 | gnl3 | 11 | 3940108 | 3951648 | protein\_coding |
| ENSDARG00000040564 | hnrnpul1 | 18 | 35826580 | 35856665 | protein\_coding |
| ENSDARG00000054823 | id3 | 17 | 27253008 | 27254905 | protein\_coding |
| ENSDARG00000019976 | idi1 | 24 | 3437586 | 3445316 | protein\_coding |
| ENSDARG00000024204 | mcm3 | 20 | 34867318 | 34880864 | protein\_coding |
| ENSDARG00000091419 | nrarpa | 10 | 10902521 | 10906093 | protein\_coding |
| ENSDARG00000044488 | pak1ip1 | 24 | 36270673 | 36287293 | protein\_coding |
| ENSDARG00000002952 | smo | 4 | 14972484 | 14981182 | protein\_coding |
| ENSDARG00000068993 | zgc:153631 | 8 | 22334670 | 22348557 | protein\_coding |
| ENSDARG00000067838 | zgc:162967 | 20 | 50212474 | 50242982 | protein\_coding |
